# One-session laparoscopic management of Mirizzi syndrome: feasible and safe in specialist units

**DOI:** 10.1007/s00464-020-07765-4

**Published:** 2020-07-06

**Authors:** Ahmad H. M. Nassar, Mahmoud K. Nassar, Ines C. Gil, Hwei J. Ng, Ahmad M. Yehia

**Affiliations:** 1 Laparoscopic Biliary Surgery Service, Department of General Surgery, University Hospital Monklands, Airdrie, ML6 0JS Lanarkshire Scotland, UK; 2 Department of Plastic Surgery, Aberdeen Royal Infirmary, Aberdeen, Scotland; 3 Department of Surgery, Centro Hospitalar de Leiria, Leiria, Portugal; 4 Faculty of Medicine, Zagazig University, Zagazig, Egypt

**Keywords:** Laparoscopic cholecystectomy, Mirizzi syndrome, Difficulty grading

## Abstract

**Background:**

To evaluate the laparoscopic management of Mirizzi syndrome, seldom diagnosed preoperatively causing difficulty when performing cholecystectomy and increasing complication risks.

**Methods:**

Analysis of a prospective single-surgeon database of 5700 laparoscopic cholecystectomies found 58 Mirizzi syndrome cases. They were managed with an intention to treat during the index admission according to protocol of single-session management of bile duct stones.

**Results:**

38/58 patients were females (65.5%). The median age was 55 years. 53 cases were emergency admissions. 34 cases (58.6%) only had ultrasound scanning. Operative difficulty was Grade IV in 34 cases (58.6%) and Grade V in 20 (34.5%) (Nassar Scale). There were 33 Mirizzi Type IA, 7 Type IB, 16 Type II and one each of Type III and Type IV. Bile duct exploration was performed in 94.8% through choledochotomy/ transfistula in 58.6% or transcystic in 36.2%. Four cases required conversion to open. Postoperative morbidity occurred in 29%. Two 30-day mortalities occurred from pneumonia in two elderly patients who were late referrals.

**Conclusion:**

Although the utilization of the laparoscopic approach in managing bile duct stones is not currently widely practiced it was safer in this series than in reported series of open surgery in Mirizzi Syndrome. The optimal approach to Mirizzi Type II is via cholecystocholedochal fistula to explore the bile duct then drain with T-tube through the fistula. It is unnecessary to perform bilioenteric bypass in majority of cases, reducing the morbidity and mortality.

The adoption of single-session laparoscopic management of cases with suspected bile duct stones has clinical and cost advantages. It obviates the need for expensive, and sometimes invasive, preoperative diagnostic and therapeutic interventions. Although not widely practiced it has become accepted by most international endoscopic surgery societies and incorporated in their guidelines as the more optimal treatment where the expertise and logistics are available.

Mirizzi Syndrome (MS) is one of the most complex pathologies that can be encountered during laparoscopic cholecystectomy. It is associated with surgical difficulty, a high conversion rate, and high risk of operative complications, particularly bile duct injury.

The term Mirizzi Syndrome was popularized after the entity was described by an Argentinian surgeon, Pablo Mirizzi in 1948 [1]. The typical abnormality is an impaction of a stone in the cystic duct, which lies parallel to the common hepatic duct resulting in its obstruction.

A simple classification was proposed by McSherry [2], suggesting the two types most commonly described; Type I resulting from extrinsic compression of the common hepatic duct by stones impacted in the cystic duct or the gallbladder infundibulum (Fig. 1) and Type II where a cholecystocholedochal fistula exists.

**Fig. 1:**
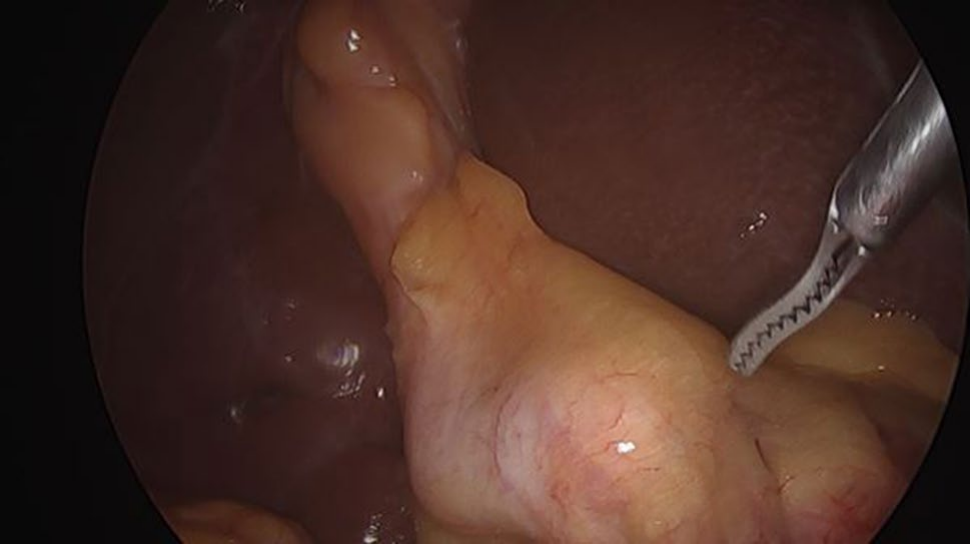
Mirizzi Type I: stone impacted in the proximal cystic duct compressing the common bile duct

Csendes [3] classified Mirizzi Syndrome into four types, further dividing Type II according to the extent of the involvement of the common hepatic duct wall in the McSherry’s Type II fistula.

## Methods

We aim to evaluate our operative management of Mirizzi Syndrome (MS). Prospectively collected data were included in a database of 5740 laparoscopic cholecystectomies (LC), including 1314 bile duct explorations carried out between February 1992 and February 2020. All cases were performed by a single surgeon. The specialized biliary firm is dedicated to managing the great majority of biliary emergencies, and almost all suspected ductal stones, admitted under surgeons, physicians, or gastroenterologists in the hospital. It receives occasional patients from other hospitals for the purpose of laparoscopic ductal exploration. 58 cases of MS were managed according to a standard protocol for suspected bile duct stones, based on single-session laparoscopic management during the index admission. Ultrasound scanning is the only routine preoperative imaging. MRCP and CT scanning are carried out occasionally and selectively, mainly when the ultrasound scan is negative for gallbladder stones in a jaundiced patient or when malignancy is suspected. ERCP is not part of our protocol for managing bile duct stones except in those unfit for surgery, in which MRCP confirms the presence of bile duct stones. All patients fit for anesthesia are prepared for theater and scheduled for surgery on the next available list. A few patients with MS associated with suspected cancer due to equivocal cross-sectional imaging and three with confirmed Mirizzi type II high up in the porta hepatis were referred to a liver surgery unit for management. These were judged to require biliary reconstruction.

Patient demographics, American Society of Anesthesiologists (ASA) classification, clinical presentation, diagnostic imaging, operative difficulty grading (Nassar scale) [4–6], presence of choledocholithiasis, gallbladder morphology, MS type, intraoperative cholangiography results, operative time, technical methods utilized, complications, readmissions, hospital stay, and the follow-up period were examined in this group.

Open access and a four-port technique is used. An attempt to display a critical view of safety is always undertaken. Hook dissection of the cystic pedicle and the use of endoclips to occlude the cystic structures are not practiced in our unit in favor of blunt dissectors and ligation of the cystic structures. Once a decision is made that a critical view of safety is not possible, alternative approaches are used including infundibular identification and dissection, transvesical access, and removal of stones or subtotal cholecystectomy as a last resort. We perform intraoperative cholangiography routinely. Mirizzi type I anomalies are suspected when there is difficulty in passing the cholangiography catheter or when cannulation is impossible. The cystic duct may be dilated and surrounded by inflammatory tissue. Choledochoscopy may occasionally have to be used to confirm the presence of stones in the intramural part of the cystic duct.

For practical purposes, the authors propose a classification into Types IA, IB (where the cystic duct is obliterated) and Types II, III, and IV. This is sufficient and includes all cases. Types III and IV are very rare and determining the exact extent of the involvement of the bile duct circumference in the cholecystocholedochal fistula in terms of percentages may not be easy to assess.

**Fig. 2:**
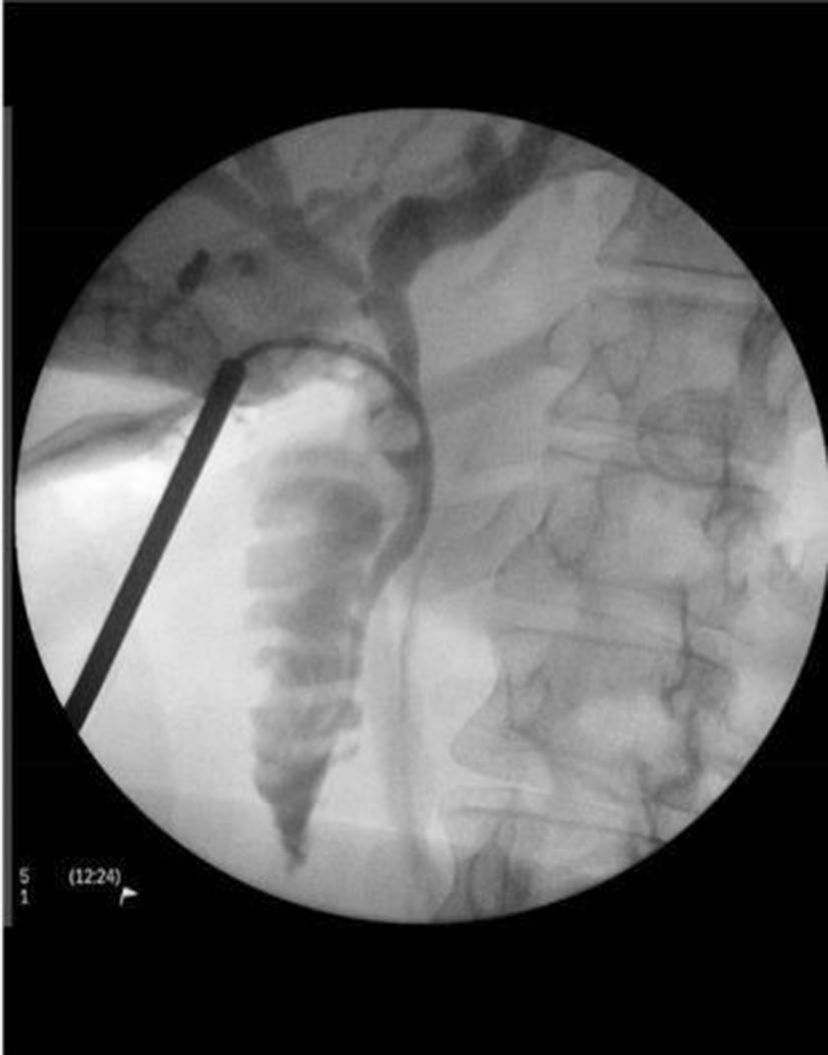
Cholangiography showing the obstructing stone in Mirizzi Type I. The narrow distal CBD demonstrates the obstacle to endoscopic stone removal

Type I stones impacted in the intramural cystic duct (Fig. 2) may be manipulated into the distal cystic duct using two instruments or dislodged using balloon catheters. Once removed, completion cholangiography or, if necessary, choledochoscopy is performed to exclude bile duct stones. The cystic duct stump is then ligated using a tie or an endo-loop. Impacted stones are fragmented mechanically, or by using ultrasound or laser lithotripsy. Repeat cholangiography and choledochoscopy are then used to exclude, and retrieve, associated bile duct stones. Should the CD be obliterated, the surgeon may occasionally decide to perform cholangiography by direct puncture of the common bile duct in order to confirm the integrity of the biliary duct system and the absence of stones within it.

Type II MS is suspected when stones are embedded in dense inflammatory tissue, when separation of an intact Hartman’s Pouch is impossible and when stones are encountered and dislodged during swab dissection of the area of the pedicle. Cholangiography is mandatory, is usually performed through the fistula and may show the presence of further stones in the bile duct.

**Fig. 3:**
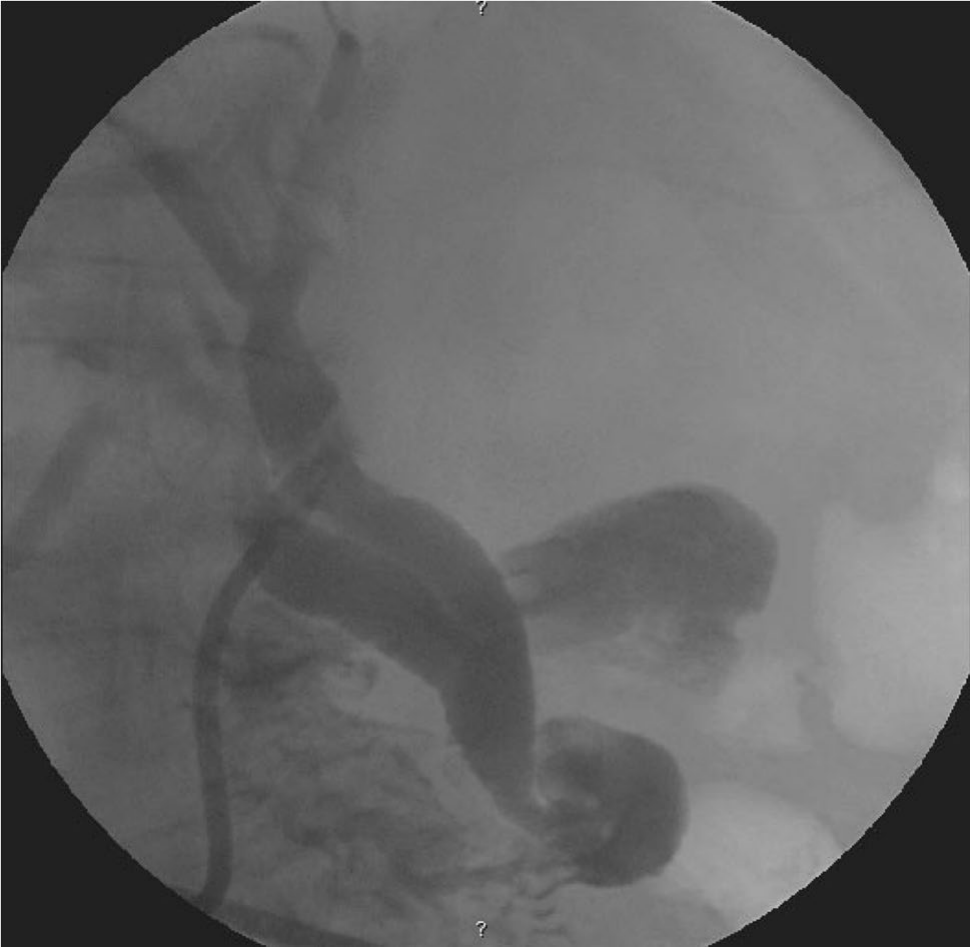
T-Tube in position after transfistula exploration and stone removal in Mirizzi Type II

In Type II MS cases, blunt swab dissection may help dislodge the offending stone or expose a cavity where the stone is impacted. Transvesical access is used to remove stones, avoid proximal dissection, and guard against ductal injury. Fragmenting and removing the stone will normally reveal the fistula and confirm the diagnosis. Cholangiography is then carried out to confirm the integrity of the bile duct and to clear any further stones through the fistula or, occasionally, through a separate choledochotomy. In our practice, mandatory biliary drainage is achieved by placing a T-Tube into the fistula with no attempts to suture or patch the fistula (Fig. 3). Bilioenteric anastomosis is not used in uncomplicated Type II MS and, in this series was only used once when the distal bile duct was very small and the area of the fistula was very fibrous, suggesting that a stricture would result from the standard approach.

**Fig. 4:**
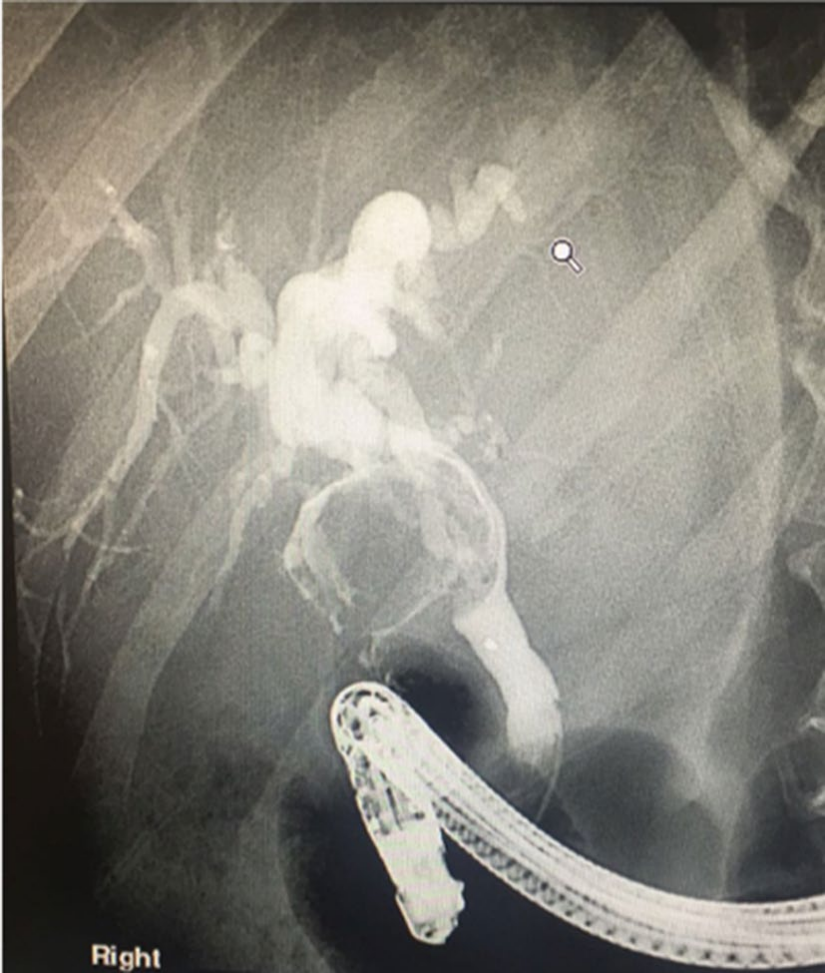
MRCP image showing the typical appearance suggestive of Types III and IV Mirizzi Syndrome abnormalities

MS Types III and IV are rarely encountered. Should a preoperative diagnosis be made (Fig. 4) consideration should be given to referring the patient to a specialist liver unit, as most patients will require biliary reconstruction.

Long-term follow-up was carried out by reviewing most patients annually at the outpatient clinic. In preparation for this paper, the available electronic records of the last 52 cases, outpatient attendances, blood tests any radiological investigations that might suggest a biliary problem, and any hospital admissions were reviewed in order to ensure complete follow-up.

No ethical approval was required as the one-session approach to bile duct stones is the standard management at our institution due to the long-term experience and availability of equipment, in line of guidelines of international societies. The consent process emphasized this and included counseling when a preoperative diagnosis of MS was available. It was recognized that endoscopic management had either already been attempted and failed before referral or was expected to fail and the fact that the only alternative would have been open surgery.

The results were analyzed with IBM SPSS 22. Chi-square test was performed on categorical variables and a *p* value of < 0.05 was considered statistically significant.

## Results

58 cases of MS were encountered (1%). Of these, 38 were females (66%). The median age was 55 years (range 29–84). 43 patients (74%) had an ASA score of 2 or 3 and 53 cases (91%) were emergency admissions.

### Clinical presentations

83% of our cases presented with current or recent jaundice and 9% with derangement of the liver function associated with episodes of severe biliary pain, cholangitis, or acute cholecystitis. Only two patients presented with acute pancreatitis associated with jaundice.

39 patients (67%) in this series had no previous admissions prior to the index presentation. 19 patients (33%) had at least one previous admission; with jaundice, acute cholecystitis or acute biliary pain, and derangement of the liver function tests. However, only two of these previous admissions were under the care of the treating biliary surgeon. They were initially treated conservatively due to a presentation with vague pain and loss of weight. They were investigated by the gastroenterologists and underwent ERCP showing impacted stones. Stents were inserted, malignancy was excluded, and the patients were referred back to the biliary firm for surgery. Eleven patients had previous admissions to other surgical or medical firms between 2 months and 2 years prior to presenting to the biliary firm. They were treated conservatively either because of uncertain diagnosis, old age, or co-morbidity. Another six patients were referred for bile duct explorations after previous admissions to other hospitals.

### Source of referral

40 patients were referred by other surgeons or physicians in the hospital and 11 by other hospitals. Only 7 were initially admitted under the care of the biliary firm.

The median interval between admission and referral to the biliary firm and the median interval from referral to surgery are shown in Table 1. More than half the patients were operated on within two days after admission and three quarters within 5 days.

**Table 1:** Demographic data, *N* = 58

Females	38 (65.5%)
Age, median (range)	55 (29–84)
ASA classification	
1	12 (20.7%)
2 or 3	43 (74.1%)
Not recorded	3 (5.2%)
Emergency admissions	53 (91.4%)
Clinical presentation	
Jaundice	48 (82.7%)
Deranged LFT^a^	5 (8.6%)
Previous admissions	19 (32.8%)
Source of referral	
Biliary firm admission	7 (12.1%)
Other surgeon	34 (58.6%)
Physicians	6 (10.3%)
Other hospital	11 (19.0%)
Days from admission to referral, mean (range)	3 (0–24)
Days from referral to surgery, mean (range)	2 (0–10)
Referral to surgery interval	
Day 0–Day 1	25 (43.1%)
Within 2 days	32 (55.2%)
Within 5 days	44 (75.9%)
More than 5 days	11 (19.0%)
Unknown	2 (3.4%)

^a^Including biliary pain, cholangitis or acute cholecystitis

### Radiological investigations

USS was the only radiological investigation in 34 cases (59%). The great majority had dilated bile ducts, the CBD diameter ranging from 9 to 20 mm. However, only one was reported to have bile duct stones and one was reported to show a suspicion of MS.

Preoperative MRCP and ERCP are not part of our protocol for patients with risk factors for bile duct stones who are fit for anesthesia. Such patients are normally prepared for laparoscopic cholecystectomy with operative cholangiography and proceed to bile duct exploration when necessary. However, in this series of MS these imaging modalities were carried out prior to referral to the biliary firm in 24 patients including 8 CT scans, 9 MRCPs, and 7 ERCPs in six patients. Only 3 CT scans, 3 ERCPs, and 3 MRCP were requested after the cases were transferred to the care of the biliary firm (Table 2).

**Table 2:** Radiological investigations

Preoperative radiological investigation, *N* = 58	
USS only	34 (58.6%)

Other imaging tests (in 24 patients)	Before referral	After referral
CT	8	3
MRCP	9	3
ERCP	7	3

Operative or postoperative investigations, *N* = 58	
Operative cholangiography	53 (91.4%)
Operative choledochoscopy	51 (87.9%)
Postoperative cholangiography	33 (56.9%)

Preoperative diagnosis of MS was only possible in 7 cases (12%) in the whole series. The rest were diagnosed at operation where the difficulty grading (Nassar Scale) [4–6] was grade IV or V in 54 (93%). The dissection of the cystic pedicle was recorded to be difficult in 56 of 58 cases. There were significant adhesions around the cystic pedicle or the porta hepatis in 45 cases and 5 patients (9%) had confirmed cholecystoduodenal fistula. When the pedicle was dissected the cystic duct was found wide in 31 cases and cystic duct stones had to be removed in 29 cases. In spite of deliberate attempts it was only possible on dissection to display the Critical View of Safety (CVS) in 19% of the cases. Other strategies were employed in the majority of cases to reduce the risk of ductal injuries including intravesical access to the neck of the gallbladder and the infundibular approach. Fundus First dissection was necessary in 14 cases.

We found Mirizzi Type IA in 33 cases, Type IB in 7, Type II in 16, Type III in one, and Type IV in one case. Mirizzi Type IA, IB, and II were significantly associated with operative difficulty Grade 4 and 5 (*p* = 0.034). Impacted stones needed blunt dissection, using a swab or the suction probe to dislodge them, and intraoperative cholangiography (IOC) confirmed the diagnosis.

Operative cholangiography was completed before ductal exploration in 53/58 cases, 91%. It failed in three Difficulty Grade 4 cases and two Grade 5 cases. In total 48 cases had CBD stones in addition to the offending stones in the cystic duct in Type I or causing the fistula in Type II. (Table 3).

**Table 3:** Operative findings, *N* = 58

Mirizzi type	
IA	33 (56.9%)
IB	7 (12.1%)
II	16 (27.6%)
III	1 (1.7%)
IV	1 (1.7%)
Cholecystoduodenal fistula (+ cholecystocolic fistula in 1)	5 (8.6%)
Difficulty grading (Nassar scale)	
I	1 (1.7%)
II	1 (1.7%)
III	2 (3.4%)
IV	34 (58.6%)
V	20 (34.5%)
Critical view of safety achieved	11 (19.0%)
Fundus first dissection	14 (24.1%)
Cystic duct stones	29 (50.0%)
CBD stones in addition to offending stone	48 (82.8%)
Operative time	
Mean [min (range)]	197 (45–420)
Median (min)	180

Type IA cases required stone removal using either transcystic exploration (TCE) (21 cases) or a choledochotomy (12 cases).

Four Type IB cases and all 16 type II cases required bile duct stone removal through choledochotomies. Choledochoscopies were performed transfistula in the Type III and Type IV cases. They had associated CBD stones removed and the patients referred to liver units for bilioenteric reconstruction.

The number of CBD stones in this series ranged from 1–17 (median 1) and their size ranged from 5 to 30 mm (median 12 mm).

Biliary drainage was established using a transcystic tube in 12 cases or a T-Tube in 28 cases. No drainage was necessary in 16 Type I cases (Table 4). Bilioenteric anastomosis was necessary in four cases: two were conversions to open surgery: one Type II case with a very fibrous pedicle and a narrowed CBD below the fistula and a Type IB case with an impacted large stone in the lower CBD. The Type III and the Type IV cases were referred to liver surgery units as they required further surgery for biliary reconstruction. Type IA, IB, and II were not significantly associated with conversion to open surgery (p = 0.0576).

**Table 4:** Operative technique, *N* = 58

Operative data	Global results	MS IA	MS 1B	MS II	MS III	MS IV
	*N* = 58	*N* = 33	*N* = 7	*N* = 16	*N* = 1	*N* = 1
Operative cholangiography	53 (91.4%)	32	5	14	1	1
CBD exploration	55	33	4	16	1	1
Transcystic	21	21	0	0	0	0
Choledochotomy/transfistula	34	12	4	16	1	1
None	3	0	3	0	0	0
Biliary drainage						
Transcystic	12	12	0	0	0	0
T-tube	28	8	3	15	1	1
None	16	13	3	0	0	0
Bilioenteric anastomosis	2	0	1	1	0	0
Conversion	2 (3.4%)	0	2	0	0	0
Bilioenteric bypass	2 (3.4%)	0	1	1	0	0
Reconstruction	2(3.4%)	0	0	0	1	1

The median operating time was 180 min with a mean operating time of 3 h and 29 min (range 45–420 min).

Abdominal drains were used in all but one of our cases. In many cases, drainage was dictated by the pathology of the gallbladder or reflected the difficulty grade. We also consider abdominal drains mandatory after choledochotomy exploration.

## Postoperative data

Postoperative morbidity occurred in 29%, including pneumonia, wound infections, urinary retention, pancreatitis, retained stones, secondary bleeding, and bile leakage. There were 10 readmissions in total (17%), two with repeat episodes. Pain after biliary drain removal caused delayed discharge between 1 and 5 days and 7 readmissions.

Reinterventions were required in six cases. Five patients underwent one or more ERCPs and reoperations were eventually necessary in three. Endoscopic interventions for retained stones were necessary in two patients with Mirizzi Type I who had impacted stones intentionally left behind for postoperative ERCP. One required 6 attempts and one had two attempts before the offending stones were removed. One MS Type I patient had a persistent right hepatic duct stricture on postoperative T-Tube cholangiography and required an ERCP. Another patient with MS Type II had persistent jaundice and bile leakage. Attempted ERCP failed due to an associated duodenal stricture and she subsequently had laparoscopic stenting of the CBD stricture. The Mirizzi Type III case initially needed an ERCP and stenting for bile leakage but appeared to have secondary hemorrhage a week postoperatively. He subsequently had a laparotomy and biliary reconstruction. The Mirizzi Type IV patient was returned on the same day to the referring liver unit where he underwent a successful biliary reconstruction the following day. Two deaths occurred as a result of pneumonia; an 81-year-old man referred 3 weeks after admission under the physicians and failed ERCP and a 78-year-old woman who died 3 weeks after uneventful surgery. None had technique-related complications (Table 5).

**Table 5:** Postoperative data, *N* = 58

Postoperative data—morbidity, *N* = 58	
Morbidity	17 (29.3%)
Surgical site infection	3 (5.5%)
Pancreatitis	1 (1.8%)
Retained stones	1 (1.8%)
Bile leakage	1 (1.8%)
Pain after drain removal	2 (3.6%)
Secondary hemorrhage	1 (1.8%)
Pneumonia	2 (3.6%)
Other	6 (10.3%)
30-day mortality	2 (3.4%)
Clavien–Dindo classification	17 (29.3%)
I	9 (15.5%)
II	4 (6.9%)
III	2 (3.4%)
IV	0
V	2 (3.4%)
Readmissions	10 (17.2%)
Pain after biliary drain removal	7 (12.1%)
Retained stones	2 (3.4%)
Acute renal failure	1 (1.8%)
Reintervention	6 (10.3%)
ERCP	3 (5.1%)
ERCP followed by reconstruction	1 (1.7%)
Reconstruction	1 (1.7%)
Reoperation for CBD stenting	1 (1.7%)

### Outcome parameters

The Median hospital stay was 11 days (3–54 days). 57% had only one hospital episode. The mean number of admissions in the series was 1.6 admissions per patient. All but two of the previous admissions were under the care of other surgeons or other hospitals before being referred to the biliary unit. The presentation to resolution period was 3 weeks on average (1–34) (Table 6).

**Table 6:** Outcome parameters

Outcome parameters, *N* = 58	
Median hospital stay, days (range)	11 (3–54)
Number of episodes, mean	1.6 per patient
One episode	33 (56.9%)
Two or more episodes	26 (44.8%)
Presentation to resolution, weeks (range)	3 (1–34)
Recurrent stones	1 (1.7%)
Long-term follow-up (range)	49 (84.4%) (3 month–13 year)

Three patients were found to have gallbladder cancer involving the cystic pedicle and infiltrating the common hepatic ducts. Preoperative diagnosis was not made as two had had negative preoperative cross-sectional imaging. The third patient was only diagnosed on histological examination with negative postoperative cross-sectional imaging, suggesting that preoperative diagnosis was unlikely. Cases with histological confirmation of gallbladder cancer are usually referred to the regional liver unit for assessment and consideration of radical surgery. No such surgery is attempted at this unit. However, all three patients in this series had associated bile duct stones, one had an empyema of the gallbladder, and one had a cholecystoduodenal and a cholecystocolic fistula with 17 large stones in the bile ducts. Postoperative staging confirmed advanced gallblader cancer infiltrating the common hepatic duct and peritoneal spread. All were judged inoperable, received palliative treatment, and died within 6 months.

Long-term follow-up was possible in 52 patients (89.6%), between 7 months and 17 years. This was either carried out by patients attending the outpatient clinics annually having been informed that follow-up was for research purposes or by reviewing the electronic hospital records periodically.

Recurrent stones occurred in only one patient 9 years after surgery. The stones were cleared endoscopically and the patient has had a further 5 years of uneventful follow-up since.

## Discussion

The surgical approach to some difficult cases of laparoscopic cholecystectomy, and whether or not open conversion becomes necessary, is usually determined by the experience of the operating surgeon and his or her ability to adapt their dissection strategy and their utilization of available instruments and techniques. MS although rare, is one of the most complex and challenging pathologies encountered during cholecystectomy. The surgeon may not, in a large percentage of cases, suspect the presence of such a severe abnormality preoperatively. Once the criteria diagnostic of MS are recognized in the course of a procedure and the diagnosis is established the surgeon would have to make decisions aiming at not only facilitating the cholecystectomy, dealing with difficult bile duct stones, and taking remedial measures for some MS types but also avoiding potentially serious complications, mainly bile duct injury.

As obstructive jaundice is an important presentation of the condition, the great majority of patients who may be affected are initially treated endoscopically in an attempt to resolve the jaundice and remove what is usually diagnosed as an obstructing bile duct stone. However, endoscopic clearance usually fails, as the offending stones are either in the cystic duct (Type I) or have already eroded the common hepatic or the common bile ducts causing a cholecystocholedochal fistula (Type II). While endoscopic stone clearance may be very difficult, endoscopic stenting and the resolution of the jaundice can usually be achieved in the majority of cases. In this study, ten patients had had ERCP preoperatively, all failing to remove the offending stones, and resorting to relieving the jaundice by stenting the CBD. In spite of that only three ERCPs made a preoperative diagnosis of MS.

A preoperative diagnosis of a Mirizzi abnormality was made in another four cases; on MRCP in three cases and ultrasound scan in one. However, a preoperative diagnosis was not made in fourteen patients who underwent cross-sectional imaging and attempted endoscopic clearance. Seven ERCPs were carried out, repeatedly in two cases, alone or in addition to nine MRCPs or eleven CT scans.

Kumar et al*.* [7] suggested that the preoperative diagnosis of MS poses a clinical challenge, as the presentation is usually similar to that of simple choledocholithiasis. Absence of jaundice as a presenting symptom (14%) could be explained by the occurrence of a fistulating variety of Mirizzi Syndrome. Greiasov et al*.* [8] reported a preoperative diagnosis rate of 27% in a large cohort of 284 patients. Following a systemic review of ten studies, Antoniou et al. [9] reported that studies with a high preoperative diagnosis rate had a significantly lower risk for conversion, procedure-related complications, and reoperation, when compared with studies with a low preoperative diagnosis rate. However, as a diagnosis of MS types II, III, or IV where cholecystobiliary fistulas exist is dependent on the extent of bile duct involvement, it is difficult to see how an accurate, if any, preoperative diagnosis can be reached and a classification made. Even where ERCP is carried out routinely, a diagnostic success rate not higher than 50% was reported [10]. All published studies have demonstrated that the limits of ERCP in MS extend only to diagnosis and stenting with no stone removal being possible.

It is clear therefore that, in spite of the perceived advantages, the incidence of preoperative diagnosis is generally low. It is much lower in our series due to our policy of proceeding to operative cholangiography and bile duct exploration in most cases of suspected bile duct stones rather than relying on preoperative endoscopic clearance. As the great majority of all MS types have associated bile duct stones other than the offending stone, choledochoscopy should be available. In our series, we used choledochoscopy in 88% of the cases.

The definitive diagnosis of MS is likely to be made intraoperatively in most cases. There should be a high index of suspicion when difficult pedicles are encountered in jaundiced patients. A wide and thickened cystic duct with or without stones can be the first sign of a Type I abnormality. Dissection around the body of the gallbladder can safely identify the neck of the gallbladder.

**Fig. 5:**
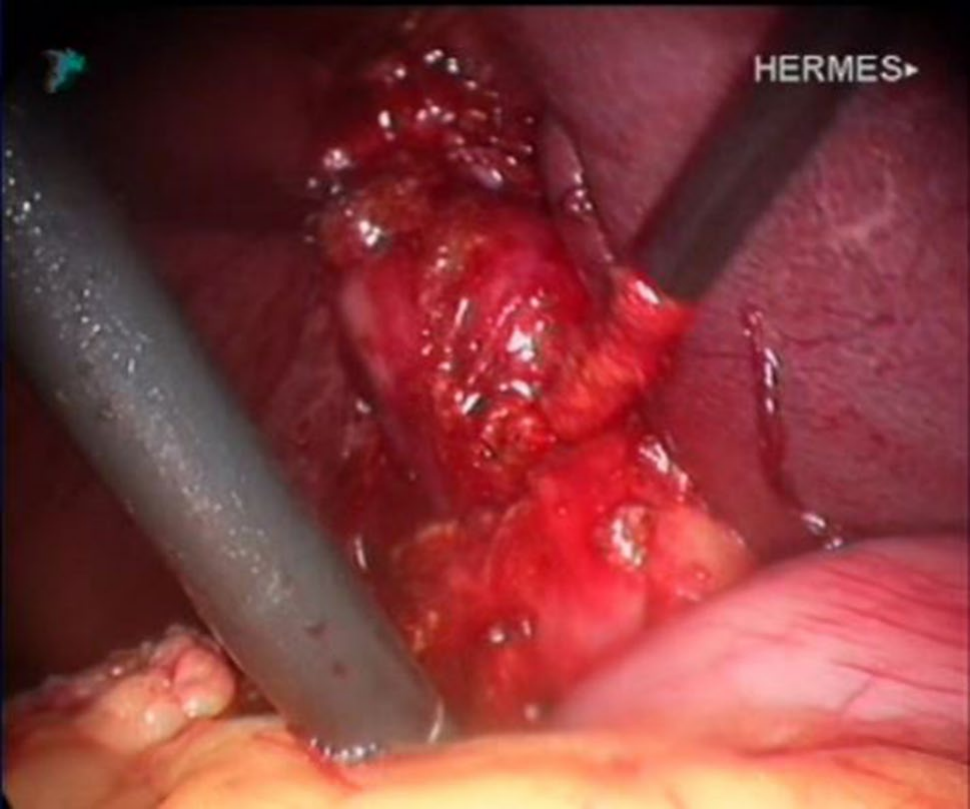
Blunt pedicle dissection with the absence of critical view of safety

Fundus first dissection was carried out in a quarter of our cases. However, in order to ensure safe dissection in the absence of a clear critical view of safety, this should not be attempted in the presence of impacted proximal stones. Removal of palpable stone/stones by opening the neck of the gallbladder can facilitate safe cystic duct dissection and help to prepare it for cholangiography. Blunt dissection is the main stay in such cases, with swab dissection being the safest method. There is no place for sharp or diathermy hook dissection of the cystic pedicle. Failure to develop the pedicle satisfactorily may indicate the presence of a Mirizzi Type II or higher. Although we routinely attempt to display the critical view of safety it was not possible to achieve that in 80% of our MS cases (Fig. 5). It is necessary to abandon this approach once a large stone is detected at the pedicle, or a very contracted gallbladder is encountered. An intravesical approach, opening the gallbladder and removing all pedicle stones is a safer strategy, preparing the pedicle for the possibility of a subtotal cholecystectomy.

Although we usually aim at a complete cholecystectomy, subtotal cholecystectomy is a valid and safe option subject to the experience of the surgeon, the MS type, and whether the condition of the tissue of the Hartman’s Pouch would allow such an approach. We consider cholangiography to be mandatory in all such cases, both to confirm bile duct integrity and to exclude CBD stones. Choledochoscopy can also help to confirm the integrity of the bile duct and to remove bile duct stones.

The difficult surgical management of MS, whether open or laparoscopic, is due to the presence of an intense fibrotic process and/or communication between the gallbladder and the common hepatic duct. The increased incidences of cholecystoenteric fistulae, adhesions, CBD stones, and gallbladder cancer determine the operative difficulty and, subsequently, surgical approach.

Rust et al. [11] suggested that MS may be a contraindication to laparoscopic cholecystectomy. However, this was a very early study (1991) and it assumed that a preoperative diagnosis is available. An intraoperative diagnosis is only confirmed once the pedicle has been dissected and, in most Type II or higher cases, the offending stone(s) are dislodged. While the remedial steps necessary may require open surgery, e.g., bilioenteric bypass, this will depend on the surgeon’s experience and skills and we do not, therefore, agree that MS is a contraindication to laparoscopic surgery. In our view an experienced biliary surgeon is able to diagnose MS and handle the difficult pedicle laparoscopically. Conversion to open surgery will result in the surgeon changing their position in relation to the patient, lose the laparoscopic view, and the moderating effect of being observed by the assistant, have their left hand obscuring most of the operating field while holding or attempting to hold the stone and, believing the stone to be in the Hartman’s Pouch/infundibulum, being tempted to dissect proximal to it. This may cause an inexperienced surgeon to open the common hepatic duct in his or her search of the cystic duct, a recognized mechanism of duct injury.

Only a few studies have been published describing experience with the laparoscopic technique for the treatment of MS. These were mostly case reports or case series. There are no randomized controlled trials comparing open and laparoscopic treatment of MS and these are difficult to conduct due to the low incidence of MS and the few centers providing laparoscopic treatment for bile duct stones.

Most published studies report high conversion rates. Chowbey et al. [12] reported a 22% conversion rate in 27 MS patients. Reviews of laparoscopic treatment of MS report a cumulative conversion rate up to 36.4% (Yeh et al. [13]) and 16% overall complication rate with bile duct injury as the most common complication [2]. Kumar et al. [7] preferred the open approach suggesting that the laparoscopic approach is associated with increased incidence of bile duct injury.

Antoniou et al. [9] claimed that the moderate technical success rate of the laparoscopic treatment of MS suggests that it cannot be recommended as a standard procedure. Their systemic review of ten studies revealed a conversion rate of 41%.

**Fig. 6:**
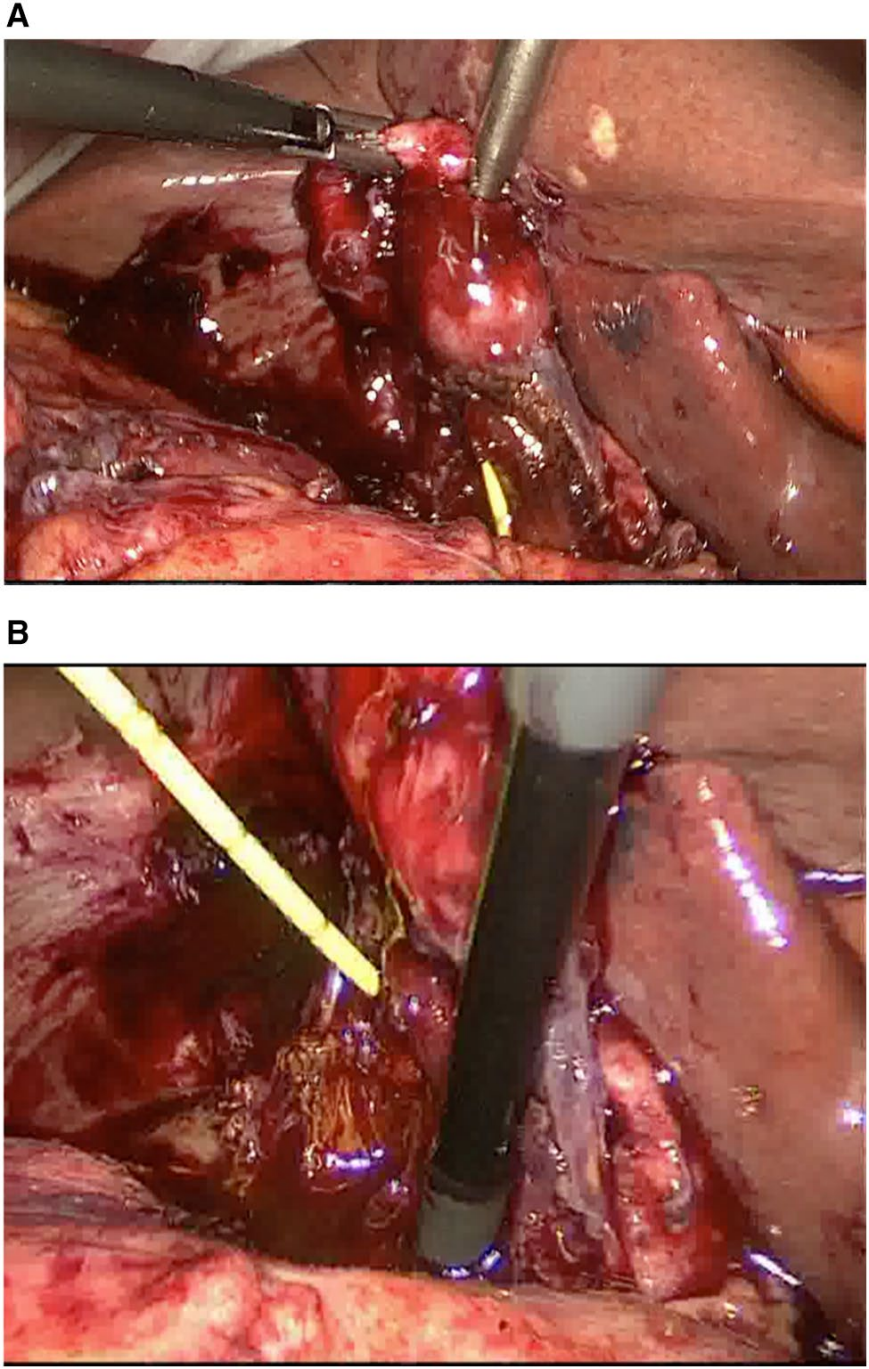
**A** Transvesical insertion of the cholangiography catheter: The catheter emerged from the proximal CBD into a Type IV Mirizzi Syndrome defect once the offending impacted stone was dislodged. **B** Mirizzi Type IV abnormality: the granulation tissue gap resulting from erosion by stone (dislodged) is seen between a cholangiography catheter in the common hepatic duct and a choledochoscope in the distal common bile duct

However, Kamalesh et al. [14] approached MS laparoscopically in 20 cases and completed the procedures in 70%, in spite of a relatively high incidence of MS Type II (40%) and Type III (20%). They recommended the use of subtotal cholecystectomy as they did in most of their patients, with the remnant of the Hartman Pouch being left and used to construct a choledochoplasty. We have resorted to subtotal cholecystectomy in only three patients, two Type I and one Type III. A transvesical approach to dissection and cholangiography may help confirm the presence of ductal defects in severe forms such as MS Type IV, once the impacted stone has been dislodged (Fig. 6A). This can then be confirmed using cholangiography and choledochoscopy (Fig. 6B).

In our view the routine use of subtotal cholecystectomy can lead to over-classifying cases as MS when in fact they had simpler impacted Hartman pouch or cystic duct stones. This “Pseudo-Mirizzi Syndrome” was suggested by Mercado et al. as a possible cause of over-diagnosis of MS and of attributing bile duct injury to a Mirizzi abnormality [15]. This occurrence will be more frequent if the surgeon does not perform cholangiography or choledochoscopy and may lead to retained CBD stones. Moreover, in the majority of Type II cases, the impacted stone and the inflamed pedicle result in friable granular tissue at the pedicle and fistula. It is therefore impossible to find any healthy tissue for a subtotal cholecystectomy or a choledochoplasty. A choledochoplasty was only attempted in our one Type III case and resulted in a bile leakage requiring ERCP and stenting. The patient eventually went on to have biliary reconstruction. However, we agree with Kamalesh et al. [14] in that biliary reconstruction is not necessary in the majority of Type II MS.

In our practice, we have used the cholecystocholedochal fistula to access the bile duct via choledochoscopy in all Type II cases, confirming its integrity and removing any further CBD stones. If an occasional additional choledochotomy is done, being necessary to remove other CBD stones, primary closure of the choledochotomy and biliary drainage by T-tube into the fistula is less invasive and safer than a biliary bypass in Type II cases. Chuang et al. [16] used the same approach and concluded that what they called laparoscopic transfistula bile duct exploration (LTBDE) was a simple and safe approach for patients with MS Type II, including Csendes Type IV. They recommended that a choledochoscope equipped with lithotripsy should be available. They also confirmed that single-port access TBDE is feasible in carefully selected cases. Long-term follow-up and large prospective randomized trials are needed to verify the findings.

## Conclusion

In Mirizzi Syndrome Type I, most obstructing stones are in the cystic duct/gallbladder neck, making endoscopic clearance very difficult if not impossible. In such cases it is only possible to relieve the jaundice by stenting the CBD with subsequent stone removal requiring surgery. One-session laparoscopic management of MS optimizes the utilization of preoperative imaging, reduces the admission to referral and surgery to resolution intervals as well as the number of treatment sessions. However, the availability of the expertise and logistical support necessary for this approach remains limited to a relatively small number of centers.

With successful definitive laparoscopic treatment in 89% in this series, we believe that MS diagnosed preoperatively or at the time of surgery, should not preclude laparoscopy. Using optimal techniques, and performed by experienced specialized surgeons, the inherent benefits of the laparoscopic approach can make it safer than open surgery. The anatomical changes specific to MS make it impossible to display a critical view of safety in the majority of cases. Alternative approaches should be employed to avoid major bile duct injury.

This series demonstrates our approach to managing MS Type II. The cholecystocholedochal fistula is used in the majority of cases to explore the bile duct, which is then drained through the fistula with a T-Tube. It is unnecessary to perform a bilioenteric bypass in the great majority of such cases, thus reducing the morbidity and potential mortality.
